# Touchscreen testing reveals clinically relevant cognitive abnormalities in a mouse model of schizophrenia lacking metabotropic glutamate receptor 5

**DOI:** 10.1038/s41598-018-33929-3

**Published:** 2018-11-06

**Authors:** Ariel M. Zeleznikow-Johnston, Thibault Renoir, Leonid Churilov, Shanshan Li, Emma L. Burrows, Anthony J. Hannan

**Affiliations:** 1 Florey Institute of Neuroscience and Mental Health, Melbourne Brain Centre, University of Melbourne, Parkville, Australia; 2 Florey Institute of Neuroscience and Mental Health, 245 Burgundy St, Heidelberg, Australia; 3 Department of Anatomy and Neuroscience, University of Melbourne, Parkville, Australia

## Abstract

Metabotropic glutamate receptor 5 (mGlu5) has been implicated in certain forms of synaptic plasticity and cognitive function. mGlu5 knockout (KO) mice and mGlu5 antagonists have been previously used to study the pathophysiology of schizophrenia as they have been shown respectively to display or induce endophenotypes relevant to schizophrenia. While schizophrenia presents with generalized cognitive impairments, the cognitive phenotype of mice lacking mGlu5 has so far only been explored using largely hippocampal-dependent spatial and contextual memory tasks. To address this, we used a touchscreen system to assess mGlu5 KO mice for pairwise visual discrimination, reversal learning, and extinction of an instrumental response requiring no discrimination. Furthermore, we tested the role of mGlu5 in working memory using the Trial-Unique Non-Matching to Location (TUNL) task utilizing pharmacological ablation. mGlu5 KO mice were impaired on discrimination learning, taking longer to reach criterion and requiring more correction learning trials. Performance on reversal learning was also impaired, with mGlu5 KO mice demonstrating a perseverative phenotype. The mGlu5 KO mice responded at a higher rate during extinction, consistent with this perseverative profile. In contrast, wildtype mice treated acutely with an mGlu5 antagonist (MTEP) showed no deficits in a touchscreen task assessing working memory. The present study demonstrates learning and memory deficits as well as an increased perseverative phenotype following constitutive loss of mGlu5 in this mouse model of schizophrenia. These findings will inform translational approaches using this preclinical model and the pursuit of mGlu5 as therapeutic target for schizophrenia and other brain disorders.

## Introduction

The metabotropic glutamate receptor 5 (mGlu5) is an excitatory post-synaptic receptor localized largely at the periphery of the active zone, where it regulates both ionotropic receptor activity and intracellular processes via G protein-linked second messenger pathways^1–3^. mGlu5 receptors are found in brain regions important for cognition, including the hippocampus, striatum, and cortex^4^ and are proposed to regulate many cognitive domains and behaviours, including spatial learning, drug discrimination learning, extinction and sensorimotor gating^5–8^. Metabotropic glutamate receptors provide a mechanism by which the function of N-methyl-D-aspartate (NMDA) glutamate receptors can be modulated^9,10^. Aberrant NMDA receptor activity is implicated in psychiatric disorders, such as schizophrenia^11^. NMDA receptor antagonists induce schizophrenia-like cognitive deficits in healthy humans and rodents, including impaired cognitive flexibility^12^. Given their effects on glutamatergic signalling, and particularly on NMDA receptor activity, mGlu5 modulators are promising agents for treatment of psychiatric disorders, particularly the currently untreatable cognitive symptoms.

To explore the role of mGlu5 in cognitive domains relevant to psychiatric illness, the effect of pharmacological inhibition or genetic ablation of receptor function on rodent behavior has been commonplace. Pharmacological disruption of mGlu5 has been shown to induce spatial working and long-term memory deficits^13–20^. However, there are problems with this approach as these findings are both occasionally inconsistent^21–23^ and the most common antagonist used, 2-methyl-6-(phenylethynyl)pyridine (MPEP), has significant off-target effects on NMDA receptors^24^. In contrast, mGlu5 knockout (KO) mice have clear long-term spatial and contextual learning and memory deficits as assessed by the Morris water maze (MWM) and contextual fear conditioning (CFC) paradigms^5–7,25^. It is thought that these impairments and other behavioural abnormalities might be a result of dysfunctional NMDA receptor signaling in these mice. These mice display a complete loss of the NMDA-receptor-mediated component of long-term potentiation in the CA1 region of the hippocampus^5,26^. In addition, mGlu5 KO mice display altered behavioural responses to the NMDA receptor antagonist MK-801 indicating that NMDA receptor function  is compromised in this mouse model^25,27^. A subset of these behavioural deficits are responsive to treatment with the clinically effective antipsychotic clozapine, supporting the predictive validity of mGlu5 KO mice in modelling schizophrenia-like behaviours^27^. Thus, both KO mice and antagonist-treated mice are used to model and explore the role of mGlu5 in cognition in health and disease.

Cognitive flexibility is the ability to inhibit previously learned responses and respond to previously irrelevant information when task parameters change, typically assessed by examining perseverative behaviour on cognitive tasks^28^. Cognitive flexibility, along with working memory (WM), is known to be impaired in schizophrenia^29,30^. Cognitive impairments in schizophrenia and other psychiatric disorders are not constrained to long-term or contextual memory, yet the cognitive phenotype of mGlu5-disrupted mice has so far been explored using largely hippocampal-dependent spatial and contextual memory tasks. Examination of cognitive flexibility on mGlu5-disrupted mice has so far only been examined in hippocampal-dependent contexts, where deficits have been demonstrated^6,7,31^. No studies have investigated working memory in mGlu5 KO mice and while a few have examined mGlu5 antagonism on working memory in rats, these suffered from potential confounding factors including off-target effects on NMDA receptors and spatial navigation impairments^14,16^.

To redress this, we sought to examine the role of mGlu5 in cognitive flexibility and working memory, using clinically translatable touchscreen operant chambers. Acquisition of two-choice visual discrimination (VD) was assessed in mice with genetic ablation of mGlu5 as a measure of basic associative learning. On completion, reward contingencies were reversed, and mice were required to respond to the previously rewarded stimulus. Perseveration during completion of this reversal task was examined as a measure of cognitive flexibility. To enhance interpretation of any changes in cognitive flexibility, mGlu5 KO mice were also assessed in an extinction task. Furthermore, to assess the role of mGlu5 on working memory, C57Bl/6 mice were trained on the trial-unique delayed non-matching to location (TUNL) task. In contrast to the previously described tasks, where the outcome measures are the acquisition curves of learning and memory over many sessions, the outcome measures for TUNL are observed after successful task acquisition, when animal performance had reached a stable plateau. Working memory was assessed in C57Bl/6 mice injected with the mGlu5 antagonist 3-[(2-methyl-1,3-thiazol-4-yl)ethynyl]pyridine hydrochloride (MTEP). MTEP was administered only during these probe sessions to acutely ablate mGlu5 function while working memory was being assessed without affecting baseline task acquisition. We hypothesized that in all cases, mGlu5 disruption would induce broad deficits across all task parameters.

## Materials and Methods

### Animals and Housing

Wild-type (WT) and mGlu5 KO male mice (Grm5tm1Rod) were generated from heterozygous breeding pairs that had been maintained past generation F10 on a C57Bl/6 background^5^. Genotypes were determined by PCR, from a tail biopsy. These mice were assessed on visual discrimination, reversal learning and extinction. A separate cohort of C57Bl/6 mice were obtained from Animal Resources Centre (Murdoch, Western Australia) after weaning at 4 weeks of age. These animals were assessed on VD and RL prior to the commencement of TUNL training, with other data from this cohort previously reported^32^.

All mice were housed in standard conditions (34 × 16 × 16 cm^3^; 3–4 mice/box) from 4 weeks of age. The holding room was maintained on a 12:12 h reversed light/dark cycle at 20 ± 1 °C. At 8 weeks of age, animals were weighed daily for five days to determine a baseline weight and subsequently maintained at 85% of their free-feeding weight (FFW), with 1 g per week added until mice reached 13 weeks of age to prevent stunting. Touchscreen pre-training began at 10 weeks of age and occurred in the dark-cycle under red light. One mouse (WT) was excluded due to a failure to acquire VD. All procedures were approved by The Florey Institute of Neuroscience and Mental Health Animal Ethics Committee and were performed in accordance with the relevant guidelines and regulations of the National Health and Medical Research Council Code of Practice for the Use of Animals for Scientific Purposes.

### Apparatus

Animals were tested in automated touchscreen-based operant systems (Campden Instruments Ltd), with instructions and event recordings managed through the software Whisker Server and ABET II. Extensive apparatus methods have been published elsewhere^33^.

### Touchscreen testing

#### Pre-training

Pre-training was conducted as described previously^32^. Over two weeks, mice were habituated to the touchscreen chambers, trained first to initiate trials and subsequently to associate nose-poking at a stimulus on the screen with a food reward (Iced Strawberry Milk, Nippy’s Ltd, Australia).

#### Visual Discrimination

Visual discrimination was conducted as described previously^32,33^. In brief, VD consisted of pairwise discrimination between a rewarded (S+) and unrewarded (S−) stimulus (contrast gratings at perpendicular orientation). The location of the S+ was pseudorandomised between trials, with no location appearing more than 3 times in a row. Designation of S+ and S− was counterbalanced within genotype groups. Touching the S+ initiated a reward sequence; whereas S− touches triggered a 5 sec timeout with the house light on, no reward provision and started a correction trial. The number of correction trials is considered a measure of perseverative behaviour as it corresponds to how long an animal takes to abandon an incorrect strategy. Correction trials consisted of representation of the previous trial until a correct response was made and were not counted towards the trial limit or number of correct/incorrect responses. Each session ended once a mouse finished 30 trials or reached a 60 min time limit, whichever came first. Criterion for the VD task was 80% correct responses for two consecutive sessions. Criteria for all tasks is listed in Supplementary Table 1.

#### Reversal Learning

The session after individual mice achieved criterion on VD they began RL. Reversal consisted of an inversion of the S+ and S− designation. All other aspects of the test were identical to VD.

#### Extinction

Extinction was conducted as described previously^34^. Once all mice had completed RL they commenced the acquisition phase of extinction, consisting of five consecutive sessions. A single stimulus (white square) was displayed in the centre of the screen, which when touched triggered a tone and food reward. Animals had to complete 30 trials within 12.5 mins. For the subsequent extinction phase, the protocol was identical except the food reward was omitted, leading the mice to gradually extinguish their response to the stimulus. Response rates to the stimulus were recorded for seven sessions.

#### Trial Unique Delayed Non-matching to Location

TUNL training: TUNL training was performed as previously described^32,35^. Each trial commenced with a sample phase, in which initiation triggered the display of a white square in one of five possible locations, which disappeared upon nose-poke. After a delay, a second initiation procedure was triggered by breaking the back infrared beam. This commenced the choice phase, in which the two stimuli were presented: one in the old (sample, incorrect) location and the other in the new (correct) location. Touching the correct location resulted in reward delivery, whereas a touch to the incorrect location resulted in a 5 sec timeout with the house light on and commencement of a correction trial.

Mice were initially trained to criterion on the maximal spatial separation level, consisting of three blank locations (S3) between the correct and incorrect stimulus. Once an individual mouse reached 70% performance for two consecutive sessions, the level of its next session was reduced to S2 and then S1. Task parameters consisted of a 2 sec delay, 15 sec ITI, 5 sec correction trial ITI, 5 sec timeout and the session finishing after 45 min or completion of 36 trials.

During Stage 2, mice continued to be trained on S1 but with the centre location added as a sample location. Mice were trained on S1 until group performance became stable and then moved collectively onto S0. When group performance stabilised the mice were moved onto probe trials. Task parameters were identical to those in Stage 1 with the exception of baseline training at a 0 s delay and the session finishing after 60 min or completion of 48 trials.

MTEP probe: Stable post-training TUNL performance is ideal for assessing acute pharmacology, unlike the acquisition curves observed in VD or RL tasks. MTEP (Tocris Bioscience, Bristol, UK) was dissolved in saline and the pH was adjusted to 7.0. Mice were given i.p. injections of 20 mg/kg MTEP or saline 10 min prior to commencement of the session during the MTEP WM probe. 20 mg/kg was chosen on the basis of previous literature suggesting this would achieve near-complete receptor occupancy for the duration of the task without inducing hyperlocomotor effects^36,37^ This dose has been previously reported to induce impairments in Pavlovian fear conditioning^31^. Mice were given alternating vehicle and MTEP injection sessions for 10 days (5 days MTEP, 5 days vehicle), counterbalanced across the housing groups.

### Data analysis

The associations between genotype, session, location and stimulus on correction trial count were examined at the trial’s level using a random effect regression model to estimate effect size, with individual animals treated as random effects. This was implemented using generalised linear and latent mixed models (GLLAMMs)^38^. These are particularly appropriate for examining touchscreen data as they enable the examination of the effects of factors on a trial-by-trial basis, providing more insight than examining an aggregate ‘percentage correct’ over a session or multiple sessions. Additionally, GLLAMMs can account for non-normally distributed data, common to measures collected by touchscreens, and missing data, which occurs when mice reach criterion for a task on different days. The adjusted effect of experimental factors (genotype, session, trial location, stimulus, drug) on standard and correction trial counts were estimated by Poisson regression as incidence rate ratios (IRRs), or by logistic regression as odds ratios (ORs) for the TUNL experiments, and the effect sizes reported together with respective 95% confidence intervals to indicate the estimates’ precision and corresponding two-tailed p-values for the hypothesis of no effect. An IRR for the effect of genotype on correction trial count (count variable) is interpreted as a factor increase in the expected count of correction trials for a mouse of a given genotype compared to a control mouse, adjusting for all other experimental factors. For example, if the IRR was 1, we would expect equal counts of correction trials in both genotype groups, while for IRRs equal to 1.6, we would expect a 60% increase in count of correction trials in the genetically modified group compared to the control group, adjusting for all other experimental factors. Perseveration indices were calculated for each animal as total correction trial number divided by the number of incorrect responses on main trials (not correction trials). Total trials to criterion, total correction trials, perseveration indices and time to initiation were compared using two-tailed Mann Whitney U-tests. All statistical analyses were conducted using STATA v13IC (StataCorp, College Station, TX, USA).

## Results

### mGlu5 genetic ablation impairs visual discrimination and reversal learning

**Figure 1:**
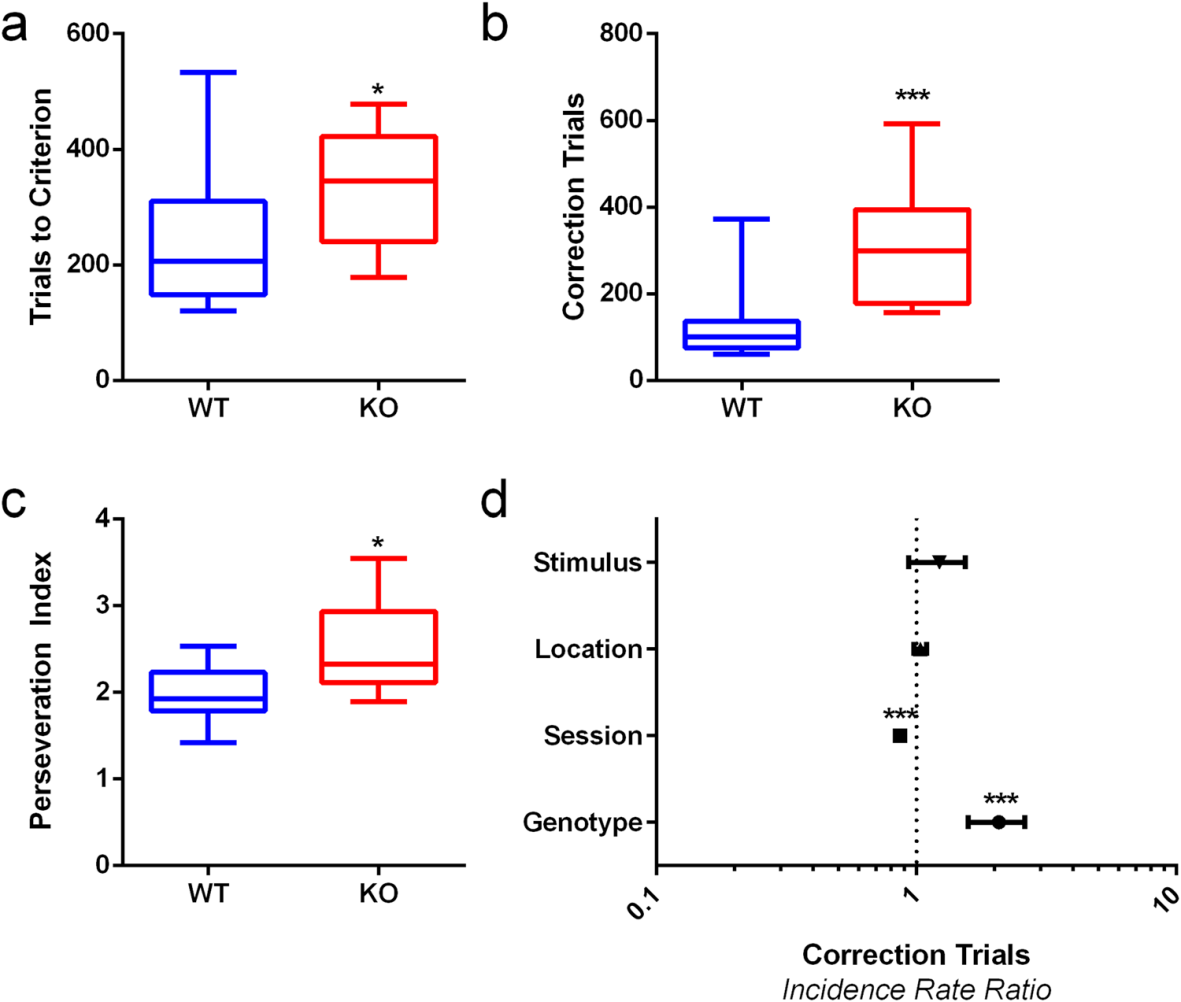
mGlu5 KO mice are impaired in visual discrimination. (**a**) The mGlu5 KO mice require more trials to reach criterion than WT littermate controls. (**b**) KO mice perform more correction trials during VD. (**c**) KO mice have an increased perseveration index (more correction trials per incorrect response). (**d**) KO mice have increased correction trial numbers per trial in VD. Correction trials reduced over sessions. There were no effects of stimulus or location bias. Panels (a–c) are expressed as box plots, with whiskers showing min-max values, box showing 25^th^–75^th^ percentile, central line corresponding to the median. Panel (d) is expressed as incidence rate ratios ± 95% CI. N = 11–12 per group; *p < 0.05, ***p < 0.001.

VD involved correctly choosing the S+ paired with the reward until the association was stable. mGlu5 KO mice took significantly more trials to reach criterion (Fig. 1(a), p = 0.030). If mice chose the S− instead of the S+ this initiated a correction trial, with a representation of the stimuli in the same locations. mGlu5 KOs had significantly more correction trials overall (Fig. 1(b), p < 0.001), when adjusted for other experimental factors (Fig. 1(d), p < 0.001, IRR = 2.035, 95% CI = 1.585, 2.614) and a greater perseveration index (Fig. 1(c), p = 0.016).

**Figure 2:**
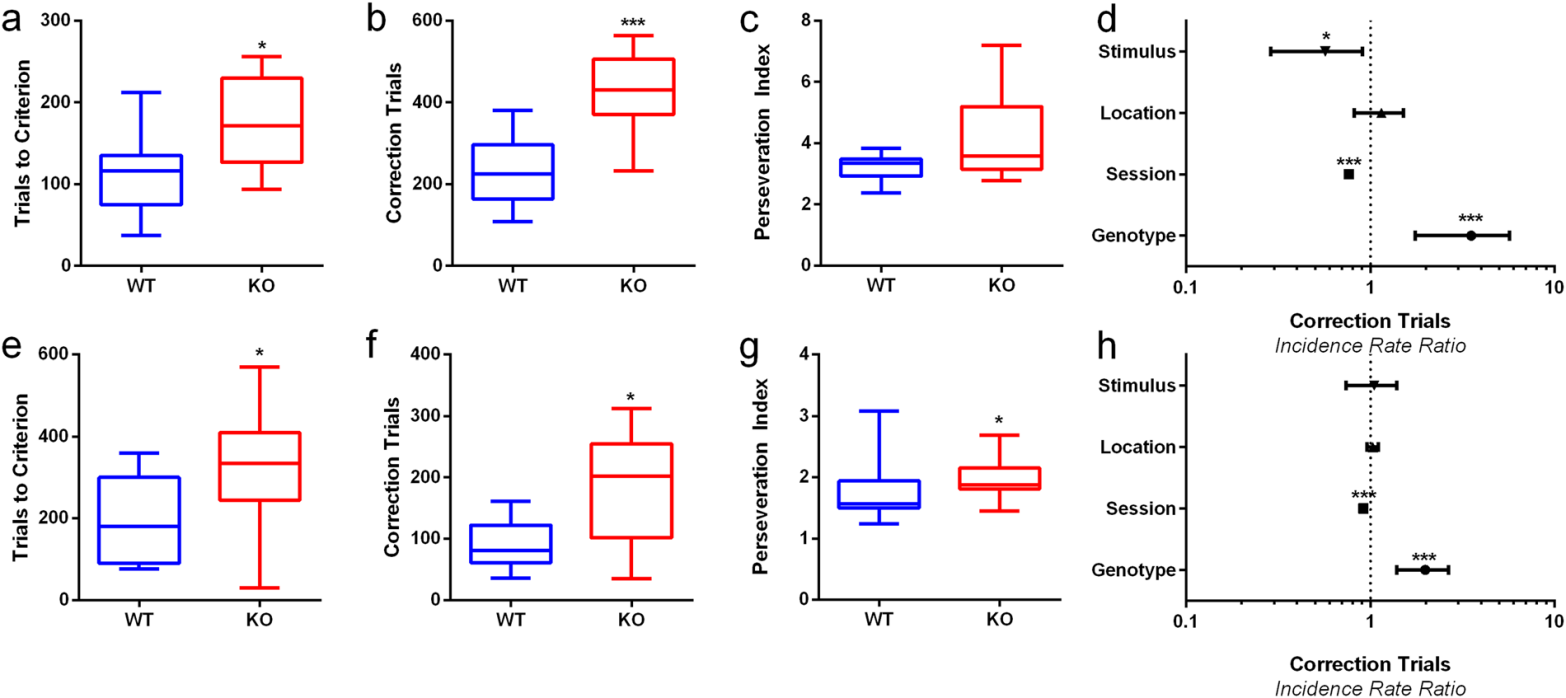
mGlu5 KO mice are impaired at reversal learning. (**a**) KO mice require more trials to reach criterion during early RL. (**b**) KO mice perform more correction trials during early RL. (**c**) KO mice do not have significantly increased correction trials per incorrect response during early RL (**d**) KO mice have increased correction trial numbers per trial in RL. Correction trials reduced over sessions. There was a small effect of stimulus bias observed. (**e**) KO mice require more trials to reach criterion during late RL. (**f**) KO mice perform more correction trials during late RL. (**g**) KO mice have significantly increased correction trials per incorrect response during late RL. (**h**) KO mice have increased correction trial numbers per trial in RL. Correction trials reduced over sessions. There were no effects of stimulus or location bias. Panels (a–c,g–e) are expressed as box plots, with whiskers showing min-max values, box showing 25^th^–75^th^ percentile, central line corresponding to the median. Panel (d and h) is expressed as incidence rate ratios ± 95% CI. N = 11–12 per group; *p < 0.05, ***p < 0.001.

After the animals had acquired stable performance on VD, the S+ and S− designations of the stimuli were switched for RL. During early RL, when performance is still under 50% correct per session, mice learn to inhibit their previously acquired S+ association^39^. mGlu5 KO mice were impaired on early RL, taking more trials to reach criterion compared to the WTs (Fig. 2(a), p < 0.001) and requiring more correction trials overall (Fig. 2(b), p < 0.001) and when adjusting for other experimental factors (Fig. 2(d), p < 0.001, IRR = 3.154, 95% CI = 1.750, 5.684). No difference was observed between WT and mGlu5 KOs in their perseverative indices (Fig. 2(c), p = 0.149), reflecting more incorrect responses but equal correction trial number per incorrect response in mGlu5 KO mice.

**Figure 3:**
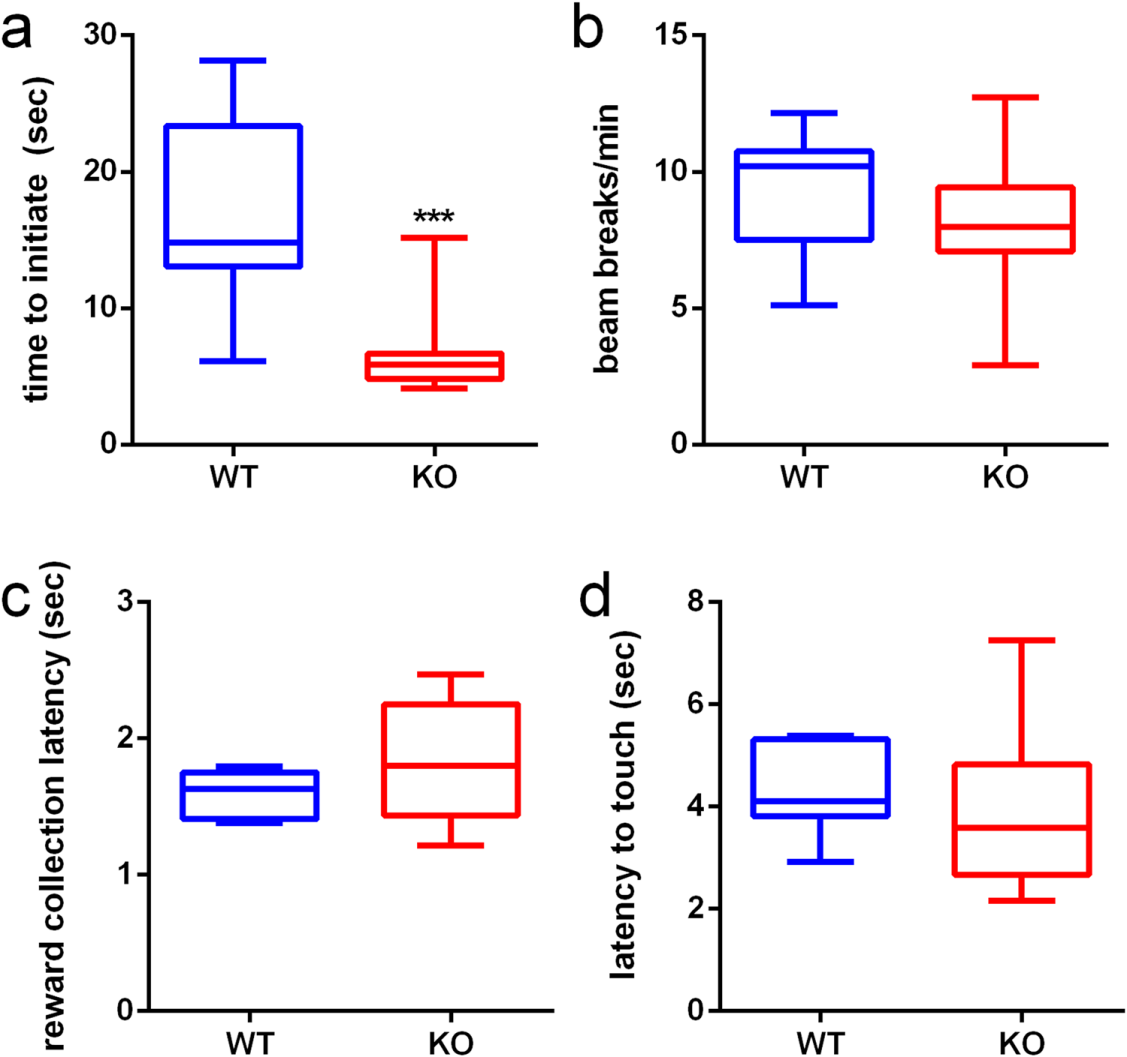
mGlu5 KO mice are faster to initiate trials during VD and RL. (**a**) KO mice are faster to initiate trials across VD and RL after the inter-trial interval has elapsed. (**b**–**d**) No differences between WT and KO mice are observed in beam breaks per minute, reward collection latency or latency to touch stimuli. Figure 4 is expressed as box plots, with whiskers showing min-max values, box showing 25^th^–75^th^ percentile, central line corresponding to the median. N = 11–12 per group; ***p < 0.001.

When mice are performing at >50% correct per session they are designated as being in late RL, where perseveration is relatively low and acquisition of the S+ improves^39^. mGlu5 KOs displayed a similar phenotype on late RL as in early RL. A significant increase in trials to reach criterion was seen (Fig. 2(e), p = 0.028) and in both overall correction trials (Fig. 2(f), p = 0.016) and when adjusting for other experimental factors (Fig. 3(h), p < 0.001, IRR = 1.917, 95% CI = 1.391, 2.644) in mGlu5 KO mice. As in VD, mGlu5 KOs had a significant increase in their perseverative index on RL (Fig. 3(g), p = 0.050).

We also observed that mGlu5 KO mice were quicker to initiate trials in both VD and RL. A significant difference was seen in median time to initiate trials across VD and RL (Fig. 3, KO: 5.86 s, WT: 14.80 s, p < 0.001). No differences between WT and mGlu5 KO mice were observed in locomotor activity, reward collection time or latency to touch stimuli across VD and RL (Fig. 3(b–d)).

### Altered extinction in the mGlu5 KO mice

**Figure 4:**
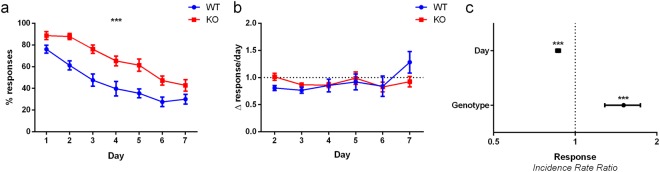
mGlu5 KO mice are impaired at extinction. (**a**,**c**) KO mice responded at a higher rate throughout the extinction sessions. (**b**) There was no difference between KO and WT mice in the rate of extinction across the sessions. Panels (a,b) are expressed as mean ± SEM. Panel (c) is expressed as incidence rate ratios ± 95% CI. N = 11–12 per group; ***p < 0.001.

During extinction, nose-poking at the stimulus is no longer coupled with a reward. Measuring the rate at which animals continue to respond provides a measure of their tendency to perseverate. mGlu5 KO mice had a higher response throughout extinction (Fig. 4(a,c), p < 0.001, IRR = 1.497, 95% CI = 1.287, 1.742). While there was a significant effect of day (Fig. 4(c), p < 0.001, IRR = 0.865, 95% CI = 0.849, 0.882), no difference was observed in the rate of extinction over days (Fig. 4(b), p = 0.977, 95% CI = 0.883, 1.137). This indicates that while both groups extinguished their responding at a similar rate over days, mGlu5 KOs have a higher baseline response rate during extinction.

### No effect of mGlu5 antagonism on working memory observed in trial-unique delayed non-matching to location task

**Figure 5:**
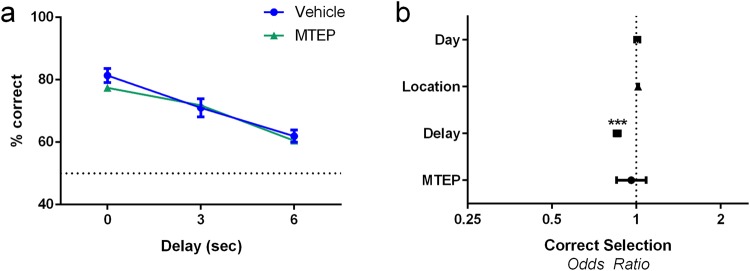
mGlu5 antagonism does not impair working memory performance. (**a**,**b**) MTEP (20 mg/kg/day for 5 sessions) has no effect on mean mouse performance or the odds of correct selection during the WM probe in wild-type mice. Panel (a) is expressed as mean ± SEM. Panel (b) is expressed as odds ratios ± 95% CI; N = 9–11 per group; ***p < 0.001.

In a separate cohort of C57Bl/6 animals, we examined the role of mGlu5 in working memory by assessing the effect of repeated administrations of the mGlu5 antagonist MTEP on working memory performance. As expected, delay had a significant effect on reducing the odds of correct selection during the TUNL working memory probe (Fig. 5(a,b), p < 0.001, OR = 0.854, 95% CI = 0.833, 0.876). No effect of mGlu5 antagonism was seen on the odds of correct selection either overall (Fig. 5(b), p = 0.508, OR = 0.960, 95% CI = 0.850, 1.084) nor in a delay x drug interaction (p = 0.528, OR = 1.017, 95%CI = 0.966, 1.070).

## Discussion

This study describes learning and memory impairments in domains previously unreported in mGlu5 KO mice. Investigating this mouse model in a touch-screen system for the first time, we demonstrate that mGlu5 KO mice have robust impairments across discrimination learning and reversal learning, as well as altered responding in extinction. We also show that pharmacological disruption of mGlu5 does not affect working memory.

This is the first study to report impairments in non-spatial pairwise discrimination learning in mGlu5 KO mice. These animals require both more standard and correction trials on a simple pairwise discrimination task, indicating non-specific cognitive impairment and suggesting that mGlu5 facilitates basic associative learning. This is similar to other cognitive impairment models demonstrating deficits in visual discrimination, such as NR2A KO mice^39^. When required to reverse their responding to the previously learnt stimuli, mGlu5 KO mice were impaired relative to WT controls. That this impairment was observed both in early reversal (<50% correct), when animals are inhibiting the previously learnt association, and late (>50% correct) reversal, when animals are learning the new association, suggests that mGlu5 KO mice have deficits in both cognitive flexibility and associative learning. This is in contrast to animals with pure deficits in either associative learning, such as NR2A KO mice^39^, or reversal learning, such as cortical NR2B KO mice^40^. This perseverative phenotype was further demonstrated by the altered extinction observed in mGlu5 KO mice. Increased responding in these animals was observed throughout the extinction period, corroborating the perseverative phenotype demonstrated during early reversal learning. The extinction alteration is consistent with impairments previously reported in mGlu5 KO mice using fear conditioning^6^ or addictive drugs^7^. Notably, we did not observe impairments of working memory in WT mice subjected to mGlu5 antagonism. This suggests that while mGlu5 may be required for task acquisition, it is not needed for working memory once mice have reached a stable performance plateau. Our finding that mGlu5 is not necessary for working memory is in contrast to previous studies identifying deficits induced by MPEP^13–16^.

The broad cognitive deficits of mGlu5 KO mice demonstrated in this present study raise the question of to what extent the impairments are due to difficulties in memory acquisition as opposed to heightened perseveration. On the one hand, the increase in total trials required for the animals to complete visual discrimination and late reversal learning are suggestive of memory acquisition problems. This would be supported by previous work showing that mGlu5 antagonists administered before (but not after) stimulus presentation hinder subsequent recall^18,31^ as well as previous studies which found cognitive impairments in mGlu5 KO mice on the acquisition of memory in the Morris water maze^5,6,25^ and in contextual fear conditioning^5,6^. However, mice with pure deficits in associative learning, such as NR2A KO mice, manifest impairments in late – but not early – reversal learning^39^. Our findings of impairments in early reversal learning and alteration of extinction, as well as increased perseverative indices and correction trial number throughout all tasks, are indicative of a perseverative profile for mGlu5 KO mice. These findings are similar to those observed in mice with cortical deletion of GluN2B, and given the role of mGlu5 in potentiating NMDA receptor activity there may be a shared mechanism^40^. Of note is the fact that we observed increases in correction trial number and perseverative indices even during visual discrimination, before the animals had learned any associations that required inhibition. Further touchscreen testing may be useful to reveal the extent of cognitive stability vs flexibility in these animals^28^. These present findings indicate that mGlu5 KO mice have both impaired memory acquisition and heightened perseveration.

Unexpectedly, we observed that mGlu5 KO mice were considerably quicker to initiate trials than WT controls. There were no differences in locomotor activity between the two groups, suggesting that this is not due to hyperlocomotion in mGlu5 KO mice. Previous studies have found no difference in motivation for a sucrose reward in mGlu5 KO mice^41^, which accompanied with our lack of observation of difference in reward collection time suggests the difference is not due to altered levels of motivation. There were also no differences in latency to touch after stimulus presentation, which we would expect if the difference in initiation time was due to increased impulsivity leading to faster responses. Previous studies have not found effects of mGlu5 antagonism on impulsivity^37,42^. However, it is possible that there are impulsivity or motivational changes in mGlu5 KO mice which could be determined with more precise testing, such as with the 5-choice serial reaction time and progressive ratio tasks.

Our observation that mGlu5 antagonism did not impair working memory is divergent with previous reports. One explanation could be that previous studies made use of the radial-arm maze or Y-maze, which require spatial navigation, while TUNL requires only spatial working memory. Thus, it is possible that the detrimental effects of mGlu5 antagonism reported in the previous studies^14,16^ may instead have been a result of MPEP impairing acquisition of a map of the environment or spatial navigation strategies, rather than affecting working memory per se. Those previous studies that used the radial-arm maze typically showed MPEP increasing reference memory errors, indicating mGlu5 antagonism induced poor acquisition of the maze. In keeping with this, MPEP has been shown to impair the stability and spatial information content of place cells^43^. It should also be noted that MPEP, in contrast to MTEP, has been shown to have off-target effects on NMDA receptors^24^. Despite MTEP having a shorter half-life than MPEP, 20 mg/kg of MTEP injected 10 min before the task commenced should have resulted in sufficient receptor occupancy throughout the session and particularly during the first half when animals complete the majority of their trials^36^. Our MTEP dosage in the present study was within the range used in previous behavioural studies^31,44^.

Given the known interaction between mGlu5 and NMDA receptors, it is worthwhile to compare our results to behavioural studies examining NMDA receptor disruption. Experiments using the NMDA receptor antagonist MK-801 show disruptions in hippocampal-dependent long-term memory^45,46^, visual discrimination^47,48^, reversal learning^46,47,49^ and extinction of both fear^50^ and appetitive^49^ memories. Specifically looking at working memory, MK-801 has been shown to impair performance^16,51–55^, but with a simultaneous decrease in performance at no delay (i.e. not in a delay-dependent manner) and frequently with hyperlocomotor side effects. Impaired performance at baseline, on other cognitive tasks, and a lack of interaction between MK-801 and delay indicates that NMDA antagonism is impairing memory formation rather than working memory per se. mGlu5 potentiation of NMDA receptors may facilitate WM in tasks requiring spatial navigation, with MPEP and MK-801 impairing performance on those tasks. In contrast, both this study and others suggest that neither mGlu5 nor NMDA receptors are necessary for WM performance in familiar environments when working memory alone is being assessed^56,57^.

More work is required to determine to what extent the cognitive impairments in mGlu5-disrupted animals are through loss of potentiation of NMDA receptors as opposed to NMDA-receptor-independent effects. In addition, it would be interesting to clarify whether NMDA-mGlu5 receptor interactions have different behavioural effects based on NMDA receptor subunit composition. For example, NR2A KO mice have both impaired discrimination and reversal learning deficits^39^, but no impairments in extinction, while loss of NR2B from different brain regions can also impair learning, with striatal loss impairing visual discrimination and cortical loss impairing reversal learning^40,58^.

The glutamatergic hypothesis of schizophrenia holds that disruption to glutamate signaling is implicated in the development of the disease. Acute administration of NMDA antagonists induce symptoms of schizophrenia in humans and animal models, and dysfunction of the NMDA receptor is hypothesized to be a key component of pathogenesis in schizophrenia^11^. mGlu5, a modulator of NMDA receptors, has altered expression in patients with schizophrenia^59,60^. Schizophrenia presents with generalized learning and memory impairments, including attention, memory and cognitive flexibility^12^. These deficits can be assessed using the CANTAB touchscreen-based cognitive assessment battery^61^. Our study has demonstrated that animals with a deficit in glutamatergic signaling display deficits in memory acquisition and cognitive flexibility on touchscreen tasks analogous to those used to assess cognition in humans suffering from schizophrenia. It should be noted that developmental absence of mGlu5 may also contribute to the observed results in the present study, which may also have relevance to the pathogenesis of schizophrenia, a neurodevelopmental disorder^62^.

In conclusion, mGlu5 KO mice constitute a robust model of learning and memory impairments and perseverative behavior, demonstrating that mGlu5 plays a more general role in cognition than just hippocampal-dependent tasks. Touchscreen operant chambers are able to assess this in a more automatable, reproducible and translatable fashion than traditional testing and have been used to observe cognitive enhancement in wild-type animals^32^. mGlu5 KO mice are cognitively impaired but still ultimately able to learn, making them a useful psychiatric disease model in which to assess putative cognitive enhancers^25,27^.

## Electronic supplementary material

Supplementary Table 1
