# Supplementary material for: Touchscreen testing reveals clinically relevant cognitive abnormalities in a mouse model of schizophrenia lacking metabotropic glutamate receptor 5
Source: Sci Rep. 2018 Nov 6;8:16412. doi: 10.1038/s41598-018-33929-3 (PMC6219561; doi:10.1038/s41598-018-33929-3)
Supplement: Supplementary file 1 — Supplementary Table 1 [file 41598_2018_33929_MOESM1_ESM.pdf]

Supplementary Information

Touchscreen testing reveals clinically relevant cognitive abnormalities in a mouse model of schizophrenia lacking metabotropic glutamate receptor 5

Ariel M Zeleznikow-Johnston[1] (B. Biomed (Hons)), Thibault Renoir[1] (Ph.D), Leonid Churilov[2] (Ph.D), Shanshan Li[1] (B. Sci), Emma L Burrows[1] (Ph.D)†, Anthony J Hannan[1,3] (Ph.D)†*

1 Florey Institute of Neuroscience and Mental Health, Melbourne Brain Centre, University of Melbourne, Parkville, Australia

2 Florey Institute of Neuroscience and Mental Health, 245 Burgundy St, Heidelberg, Australia

3 Department of Anatomy and Neuroscience, University of Melbourne, Parkville, Australia.

∗Corresponding author: Prof. Anthony Hannan, Florey Institute of Neuroscience and Mental Health, Melbourne Brain Centre, University of Melbourne, VIC 3010, Melbourne, Australia.

Email address: anthony.hannan@florey.edu.au

† Joint last authors

Grouping	Task	Criterion
**Pretraining**	Habit 2	-
	Initial Touch	30 trials in 60 minutes
	Must Touch	30 trials in 60 minutes
	Must Initiate	30 trials in 60 minutes
	Punish Incorrect	23/30 trials correct (77%) in 60 minutes for two consecutive sessions
**Visual Discrimination**	Visual Discrimination	24/30 trials correct (80%) in 60 minutes for two consecutive sessions
**Reversal Learning**	Reversal Learning	24/30 trials correct (80%) in 60 minutes for two consecutive sessions
**Trial-Unique Delayed Non-Matching To Location**	Stage 1 - Separation Level 3 (S3)	26/36 trials correct (70%) in 45 minutes for two consecutive sessions
	Stage 1 - S2	26/36 trials correct (70%) in 45 minutes for two consecutive sessions
	Stage 1 - S1	26/36 trials correct (70%) in 45 minutes for two consecutive sessions
	Stage 2 - S1	Stable group performance
	Stage 2 - S0	Stable group performance
	Probes	-
**Extinction**	Acquisition	30 trials in 12.5 minutes
	Extinction	-

Supplementary Table 1. Criteria for the touchscreen task battery
